# Mycoprotein reduces energy intake and postprandial insulin release without altering glucagon-like peptide-1 and peptide tyrosine-tyrosine concentrations in healthy overweight and obese adults: a randomised-controlled trial

**DOI:** 10.1017/S0007114516001872

**Published:** 2016-07-28

**Authors:** Jeanne H. Bottin, Jonathan R. Swann, Eleanor Cropp, Edward S. Chambers, Heather E. Ford, Mohammed A. Ghatei, Gary S. Frost

**Affiliations:** 1 Nutrition and Dietetics Research Group, Department of Medicine, Division of Diabetes, Endocrinology and Metabolism, Imperial College London, Hammersmith Campus, London W12 0NN, UK; 2 Division of Computational and Systems Medicine, Department of Surgery and Cancer, Imperial College London, South Kensington Campus, London SW7 2AZ, UK; 3 Division of Diabetes, Endocrinology and Metabolism, Department of Medicine, Imperial College London, Hammersmith Campus, London W12 0NN, UK

**Keywords:** Mycoprotein, Energy intake, Appetite hormones, Overweight, Obesity, Gastric emptying, Metabonomics

## Abstract

Dietary mycoprotein decreases energy intake in lean individuals. The effects in overweight individuals are unclear, and the mechanisms remain to be elucidated. This study aimed to investigate the effect of mycoprotein on energy intake, appetite regulation, and the metabolic phenotype in overweight and obese volunteers. In two randomised-controlled trials, fifty-five volunteers (age: 31 (95 % CI 27, 35) years), BMI: 28·0 (95 % CI 27·3, 28·7) kg/m^2^) consumed a test meal containing low (44 g), medium (88 g) or high (132 g) mycoprotein or isoenergetic chicken meals. Visual analogue scales and blood samples were collected to measure appetite, glucose, insulin, peptide tyrosine-tyrosine (PYY) and glucagon-like peptide-1 (GLP-1). *Ad libitum* energy intake was assessed after 3 h in part A (*n* 36). Gastric emptying by the paracetamol method, resting energy expenditure and substrate oxidation were recorded in part B (*n* 14). Metabonomics was used to compare plasma and urine samples in response to the test meals. Mycoprotein reduced energy intake by 10 % (280 kJ (67 kcal)) compared with chicken at the high content (*P*=0·009). All mycoprotein meals reduced insulin concentrations compared with chicken (incremental AUC_low_ (IAUC_low_): −8 %, IAUC_medium_: −12 %, IAUC_high_: −21 %, *P*=0·004). There was no significant difference in glucose, PYY, GLP-1, gastric emptying rate and energy expenditure. Following chicken intake, paracetamol-glucuronide was positively associated with fullness. After mycoprotein, creatinine and the deamination product of isoleucine, *α*-keto-*β*-methyl-*N*-valerate, were inversely related to fullness, whereas the ketone body, *β*-hydroxybutyrate, was positively associated. In conclusion, mycoprotein reduces energy intake and insulin release in overweight volunteers. The mechanism does not involve changes in PYY and GLP-1. The metabonomics analysis may bring new understanding to the appetite regulatory properties of food.

In the present context of the increasing worldwide prevalence of obesity and type 2 diabetes mellitus (T2DM), there is a need to understand the impact of food products on appetite regulation and glycaemic control in overweight and obese people. Of the three macronutrients, protein is recognised as the most potent appetite suppressor both in rodents and in humans^(^ ^1^ ^–^ ^3^ ^)^. Research in the past two decades has shown that protein-rich loads increase satiety and lead to decreased energy intake acutely^(^ ^2^ ^,^ ^4^ ^–^ ^7^ ^)^. Studies have demonstrated that protein supplementation increases weight loss and limits both fat-free mass loss and the decrease in energy expenditure, which normally occurs during weight loss by energy restriction^(^ ^8^ ^–^ ^11^ ^)^.

Increasing dietary fibre intake has also been associated with reduced energy intake^(^ ^12^ ^–^ ^15^ ^)^. In particular, soluble viscous fibres are thought to delay gastric emptying, slow nutrient absorption, and increase the production of anorectic gastrointestinal (GI) hormones, glucagon-like peptide-1 (GLP-1) and peptide tyrosine-tyrosine (PYY), through bacterial fermentation and production of SCFA^(^ ^16^ ^–^ ^19^ ^)^.

Quorn™ products are vegetarian meat replacements commonly consumed in the UK. The main ingredient of Quorn™ is mycoprotein, which is the RNA-reduced biomass produced from the continuous fermentation of the filamentous fungus *Fusarium venenatum*. Mycoprotein, as used typically, contains 25 g of solids, including 11 g of protein and 6 g of fibre/100 g (data from Marlow Foods Ltd). The fibre content is attributed to the cell wall and is composed of 2/3 branched 1–3 and 1–6 *β*-glucan and 1/3 chitin, creating a fibrous chitin–glucan matrix with low water solubility (88 % insoluble). This fibrous glucan–chitin complex is specific to fungal mycelium and not frequently present in human food.

Owing to its relatively high protein and fibre content, mycoprotein presents an attractive food product to improve appetite regulation and postprandial glycaemic and insulin responses in overweight and obese individuals at risk of developing T2DM. Previous studies in lean individuals have found that mycoprotein reduces postprandial glucose and insulin concentrations^(^ ^20^ ^)^, and energy intake at a subsequent meal^(^ ^21^ ^,^ ^22^ ^)^. There has only been one study in overweight volunteers that did not show any reduction in energy intake at 4 h^(^ ^23^ ^)^. However, this study used half of the portion of mycoprotein found in commercially available mycoprotein products. Given previous observations, there is a need to ascertain whether mycoprotein exerts a dose-dependent effect on energy intake and glycaemic control in overweight and obese individuals and to determine the mechanism through which mycoprotein may exert its action. To the best of our knowledge, there are no published studies investigating the effect of mycoprotein on GI hormones.

The primary purpose of the present study was to investigate the effect of low, medium, and high doses of mycoprotein on energy intake and glucose homeostasis compared with isoenergetic and protein-matched chicken meals in overweight and obese individuals. We hypothesised that mycoprotein would reduce acute energy intake in overweight and obese volunteers compared with a macronutrient-matched chicken meal in a dose-dependent manner by delaying gastric emptying and increasing the release of PYY and GLP-1.

## Methods

We undertook two randomised single-blinded controlled trials to investigate the impact of mycoprotein (nutritional information in Table 1) on appetite regulation. Part A investigated the effect of three levels of mycoprotein compared with chicken on appetite, acute and 24-h energy intake, glucose and insulin concentrations, and PYY and GLP-1 concentrations. Part B investigated the effect of the highest content of mycoprotein used in part A compared with chicken on appetite, glucose and insulin concentrations, gastric emptying, and energy expenditure and substrate oxidation.

**Table 1:** Nutritional composition of mycoprotein

Per 100 g of mycoprotein	
Energy (kJ)	356
Energy (kcal)	85
Protein (g)	11·0
Available carbohydrate (g)	2·0
Dietary fibre (g)	6·0
Fat (g)	3·0
MUFA (oleic acid) (g)	0·3
PUFA (g)	1·4
Linoleic acid (C18 : 2) (g)	1·0
*α*-Linolenic acid (C18 : 3) (g)	0·4
SFA (g)	0·4
Palmitic acid (C16 : 0) (g)	0·3
Stearic acid (C18 : 0) (g)	0·1

### Subjects

Overweight and obese volunteers aged 18–65 years with a BMI of 25–32 kg/m^2^ were recruited to take part in this randomised controlled single-blinded study. This study was conducted according to the guidelines laid down in the Declaration of Helsinki, and all procedures involving human subjects were approved by the Hammersmith and Queen Charlotte’s Research Ethics Committee (Ref no. 09/H0707/51). Written informed consent was obtained from all subjects. The trial was registered on www.clinicaltrials.gov (NCT02053025). Travel expenses to the research centre were reimbursed and participants were compensated for their involvement based on their participation rate.

### Recruitment

Participants were recruited via posters placed around Imperial College campuses in London, UK and internet advertising. Potential participants were invited for a screening visit to check eligibility at the National Institute for Health Research (NIHR)/Wellcome Trust Clinical Research Facility at Imperial College Healthcare NHS Trust, hereafter referred to as the research centre. Anthropometric measurements, medical history and haematological and biochemical blood screen were collected. To be included, participants had to be overweight or obese with a BMI of 25–32 kg/m^2^ but otherwise healthy (no medication or chronic disease), aged 18–65 years, with no history of eating behaviour disorder. Exclusion criteria included a history of alcoholism or substance abuse within the past 12 months; smoking; a medical or psychological condition that would interfere with the ability to participate in the study; women who were pregnant or breast-feeding or had a pregnancy within the last year; and participation in another clinical trial or blood donation within 3 months of study commencement. For part A, participants were asked to taste three *ad libitum* commercially available meals, which were similar in energy content and composition, and asked to choose their favourite.

### Sample size calculation

The sample size was calculated on the primary outcome, a decrease in energy intake, using the study by Burley *et al*.^(^ ^21^ ^)^. Following an isoenergetic meal containing 27 g of protein from mycoprotein or chicken, they reported a significant 18 % difference in energy intake with an sd of 16. With a level of statistical significance set at *α*=0·05 and a power of 90 %, the sample size *n* needed for this study was 27. To account for a 30 % dropout, a minimum of thirty-five participants were recruited to take part in the appetite study.

### Visit randomisation and preparation

All eligible participants attended a practice visit at the research centre in order to acclimatise to the protocol and the research environment. The order of the treatment visits was randomised for each eligible participant following the screening visit using a randomising web-based programme (www.random.org). In addition to this acclimatising visit, part A included six visits and part B included two visits. Participants did not need to complete both parts to be included in the study. Visits were separated by a washout period of 3–7 d. Participants were asked to refrain from drinking alcohol and to avoid any strenuous exercise for 24 h before each visit. They were also asked to consume the same evening meal and fast for 12 h before arriving for their visit to limit variability at baseline.

### Visit design

**Fig. 1:**
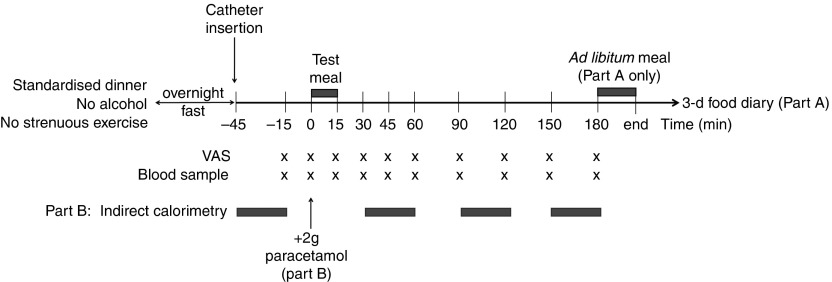
Protocol of the study visits. Participants arrived at 08.30 hours in a fasted state. The test meal consisted of a standardised mycoprotein or chicken risotto. The *ad libitum* meal was consumed 180 min after the test meal until the participant was fully satisfied. Paracetamol was given in part B as a surrogate measurement of gastric emptying. VAS, visual analogue scale.

The sequence of events during each visit for both part A and B is detailed in Fig. 1. Following a 12-h overnight fast, volunteers were asked to arrive at the research centre at 08.30 hours. After the insertion of the catheter, two fasting blood samples were taken, following which volunteers were asked to consume the test meal within 15 min. Volunteers were blinded to the meal type. Fasting and postprandial blood samples were collected at regular time points over 3 h. Subjective hunger, nausea, desire to eat, prospective food intake and fullness were assessed at the same time points using visual analogue scales (VAS). Palatability of the test meal was assessed at 15 min following consumption. VAS were analysed by measuring the distance in millimetres from the null extremity to the mark.

Plasma samples for GLP-1 and PYY analysis were collected into lithium heparin tubes containing aprotinin (Trasylol, 20 μl/ml of whole blood; Nordic Pharma) on ice and immediately centrifuged and separated. Serum samples for glucose and insulin analysis were left to coagulate at room temperature for 15 min before being centrifuged and separated. All samples were stored at −20°C until analysis at the end of the study.

### Part A: appetite, energy intake, gastrointestinal hormones and glucose homeostasis study

#### Test meal

A 350 g risotto containing low (44 g), medium (88 g) or high (132 g) contents of mycoprotein or equivalent amounts of protein from chicken was served in a randomised order on six separate occasions (Table 2). All meals were designed to closely match for energy content. Mycoprotein and chicken meals at each protein content were designed to closely match for energy and macronutrient content. Table 2 presents the mean energy and macronutrient content of the meals obtained by two nutritional analyses carried out by independent nutritional companies. Vegetables, rice, water, cheese, oil and pesto were used to achieve weight and macronutrient matching. The main difference in nutritional composition resulted from the fibre content, which was not matched between meals.

**Table 2:** Quantities of mycoprotein and chicken and nutritional composition of the test meals

	Quantities		Nutritional composition per serving					
	MP	C	Energy (kJ)	Energy (kcal)	CHO (g)	Fat (g)	Protein (g)	Fibre (g)
High MP	132	−	1862	445	26	8	37	10
High C	−	66	1774	424	25	9	40	3
Medium MP	88	−	1820	435	26	9	31	8
Medium C	−	44	1749	418	29	8	30	3
Low MP	44	−	1749	418	28	9	24	6
Low C	−	22	1703	407	30	9	23	4

MP, mycoprotein; C, chicken; CHO, carbohydrates.

#### Assessment of energy intake

At the end of the 3-h period, participants were served the meal that they had chosen during the screening visit. This *ad libitum* meal was served in excess, and participants were asked to eat until they were comfortably full. Participants were isolated during this feeding part of the study. The *ad libitum* meal was weighed before and after, and energy intake was calculated from the manufacturer’s nutritional information. Participants were then free to leave the unit but were asked to keep a detailed record of their food intake over the following 24 h. Volunteers were asked to provide details regarding the method of cooking, the quantities using measurements such as cups or tablespoons, the detail of the brands and specific details about the food (packaging for commercial meals). Instructions and examples were provided to the participants. Any unclear recording was further clarified with the participant. The food questionnaires were analysed by an independent researcher trained in nutritional analysis. Food portion sizes were estimated using Food Standards Agency portion size references, and energy and macronutrient intake were calculated using DietPlan 6.50 (Forestfield Software Ltd).

#### Blood samples

Serum concentrations of glucose were measured by enzymatic method at the end of the study using an Abbott Architect ci8200 analyzer in the Department of Biochemistry at Hammersmith hospital. Insulin-like immunoreactivity was measured by RIA using a Millipore Human Insulin Specific RIA Kit (Millipore Corporation) according to the manufacturer’s specified protocol. PYY-like and GLP-1 immunoreactivity was measured with an established in-house RIA^(^ ^24^ ^,^ ^25^ ^)^. Samples for insulin, PYY and GLP-1 analyses were assayed in duplicate. For the PYY RIA, the antibody cross-reacted fully with the biologically active circulating forms of PYY (PYY_3–36_ and PYY_1–36_) but not with pancreatic polypeptide or other known GI hormones. The detection limit of the PYY assay is 2·5 pmol/l, and the reported in-house intra- and inter-assay variations are 5·8 and 9·8 %, respectively. For the GLP-1 RIA assay, the antibody cross-reacted 100 % with all amidated forms of GLP-1, but did not cross-react with glycine-extended forms (GLP_1–37_ and GLP_7–37_) or any other known pancreatic or GI peptide. The limit of detection is 7·5 pmol/l, and the reported in-house intra-assay and inter-assay variations are 5·4 and 11·5 %, respectively.

Fasting baseline measurements were averaged to obtain a unique baseline value before statistical analysis.

#### Insulin sensitivity

Postprandial insulin sensitivity was estimated using the Matsuda Index^(^ ^26^ ^)^, *β*-cell output was estimated using the insulinogenic index^(^ ^27^ ^)^ and *β*-cell function in the context of insulin resistance was estimated using the disposition index^(^ ^28^ ^)^.

### Part B: gastric emptying, energy expenditure and substrate oxidation study

#### Test meal

The same high-mycoprotein meal (132 g) risotto or matched chicken meal from part A was served in a randomised order on two separate occasions. The test meal was served with 2 g of paracetamol dissolved in 250 ml of water to assess gastric emptying. Participants were asked to consume the food and drink within 15 min and at the same rate on both occasions.

#### Indirect calorimetry

Upon arrival in the morning, fasted participants were asked to void. Throughout their visit, they were asked to collect all urine into a container and to void again into the container at the end of the visit for the measurement of urinary N excretion from urea. The total time of urine collection was recorded. Resting energy expenditure (REE) was measured at baseline and every hour for 3 h by open-circuit indirect calorimetry (Gas Exchange Monitor; GEM Nutrition). Before each measurement, the calorimeter was calibrated with ‘zero’ (0·00 % O_2_, 0·00 % CO_2_) and ‘span’ (20·00 % O_2_, 1·00 % CO_2_) gases (BOC Gases). The volunteers were asked to lie in a semi-recumbent position under the canopy and were allowed to watch television, read or listen to music. The measurements were first allowed to stabilise for 10–15 min, following which the VO_2_ and VCO_2_ were recorded every minute for 15 min. The mean of the last 10 VO_2_ and VCO_2_ measurements was calculated; any value exceeding mean (sd 2) was excluded. No buffet meal was served at the end of the study.

#### Resting energy expenditure and substrate oxidation

REE was estimated from VO_2_ and CO_2_ production in a given time using the following equation by Weir^(^ ^30^ ^)^. Measurements of VO_2_ and CO_2_ production were used to quantify substrate oxidation in the body^(^ ^31^ ^)^.

#### Blood samples

Serum concentrations of glucose and paracetamol were measured by enzymatic method at the end of the study using an Abbott Architect ci8200 analyzer (Abbott Diagnostics) in the Department of Biochemistry at Hammersmith hospital. Cumulative paracetamol concentrations were used to calculate percentage paracetamol absorption from 0 % (at *t*=0) to 100 % (at 180 min) and percentage gastric emptying (from 100 to 0 %). Each individual gastric emptying curve was adapted to a third-degree polynomial. The T50 (time to reach 50 % of gastric emptying) was interpolated from the non-linear fit, as described in a previous study by Näslund *et al.* ^(^ ^29^ ^)^. Insulin-like immunoreactivity was measured by RIA using a Millipore Human Insulin Specific RIA Kit according to the manufacturer’s specified protocol. Fasting baseline measurements were, similarly to part A, averaged to obtain a unique baseline value before statistical analysis.

### Statistical analysis

Results are presented as mean values and standard deviations or as geometric mean with 95 % CI of the mean. All results were initially analysed by repeated-measures linear mixed model. The main effects included protein type (chicken *v.* mycoprotein), protein content (low, medium, high), as well as time for plasma and serum samples and appetite ratings. Interactions between protein type and content and protein type and time were included in the final model when significant. Significant interactions are mentioned in the results section when significant only. Relevant covariates, such as sex, age and BMI, were included in the initial model and removed if their effect was not significant. For time-profile variables, the baseline value was included as a covariate in order to correct for baseline differences, and correlation matrices of residuals were chosen by assessing the model by −2 log likelihood. For each model, the distribution of the residuals was checked for normality. The homoscedasticity of the residuals was checked by plotting the observed values against the predicted values. When the mixed model was not validated, data were log-transformed and analysed by mixed model or analysed directly by non-parametric tests, as described. All *post hoc* tests were carried out using Bonferroni’s corrections. Statistical analysis was performed on SPSS version 21 (IBM Corporation).

### ^1^H NMR spectroscopy-based metabonomic analysis

^1^H NMR spectroscopic profiles were obtained from plasma and urine samples collected from volunteers involved in parts A and B, respectively. Samples were prepared using the protocols outlined by Beckonert *et al.* ^(^ ^32^ ^)^. All spectroscopic analyses were performed on a 700 MHz Bruker NMR spectrometer, operating at 300 K and equipped with a 5 mm ^1^H(^13^C/^15^N) inverse cryoprobe. For each urine sample, a standard one-dimensional NMR spectrum was acquired with water peak suppression using a standard pulse sequence. For each spectrum, eight dummy transients and 128 transients were collected into 64 K data points with a spectral width of 12·001 parts per million. For the plasma samples, water-suppressed Carr–Purcell–Meiboom–Gill spin-echo spectra were recorded. In this experiment, eight dummy transients were followed by 128 transients and collected in 64 K data points. ^1^H NMR spectra were manually corrected for phase and baseline distortions. Urine samples were referenced to the trimethylsilylpropionic acid (TSP) singlet at *δ* 0·0, and plasma samples were referenced to the anomeric proton of *β*-glucose at *δ* 5·223. Spectra were digitised using an in-house MATLAB (version R2009b; The Mathworks, Inc.) script. To minimise baseline distortions arising from imperfect water saturation, the region containing the water resonance was excised from the spectra. Orthogonal projection to latent structures-discriminant analysis (OPLS-DA) was performed in MATLAB using scripts provided by Korrigan Sciences Ltd. Here, the spectroscopic profiles served as the descriptor matrix, and class membership (chicken and mycoprotein diet) was used as the response variable. Further OPLS models were constructed using volunteer metrics (fat-free mass) and outcome measures (fullness and hunger) as continuous response variables to illuminate associated metabolic variation. Correlation coefficients plots were constructed from the model outputs by back-scaling transformation to display the contributions of each metabolite to sample classification. Colour represents the significance of correlation for each metabolite to class membership. The predictive ability (Q^2^Y) of each model was calculated using a 7-fold cross-validation approach, and model validity was established by permutation testing (1000 permutations).

## Results

### Volunteers

Part A of the study was completed between August 2010 and September 2011. Part B of the study was completed between April 2012 and September 2012. A total of 293 volunteers contacted the research team, ninety-two were screened and sixty-six were randomised into the study. A total of sixteen participants withdrew from the study for various reasons (dislike of the meals, dislike of the protocol, repeated difficulties with catheter insertion or blood sampling, unplanned pregnancy).

### Part A: appetite, energy intake, gastrointestinal hormones and glucose homoeostasis study

#### Volunteers

A total of thirty-six volunteers (age 33 (sd 14) years, BMI 28·1 (sd 2·3) kg/m^2^) completed the study. Baseline characteristics are shown in Table 3.

**Table 3:** Baseline characteristics of volunteers (Mean values and standard deviations)

	Part A		Part B	
	Mean	sd	Mean	sd
Age (years)	33	14	37	18
Sex	19 males/17 females		5 males/9 females	
Height	168·5	9·5	170·0	8·5
Weight (kg)	80·2	12·0	82·7	12·5
BMI (kg/m^2^)	28·1	2·3	28·4	2·5
Ethnic origin				
White	20		5	
Asian	6		2	
African	3		5	
Other	7		2	
Fat mass (kg)			26·9	9·0
Fat-free mass (kg)			54·9	11·3
Body fat (%)			32·7	8·6

#### Appetite ratings

**Fig. 2:**
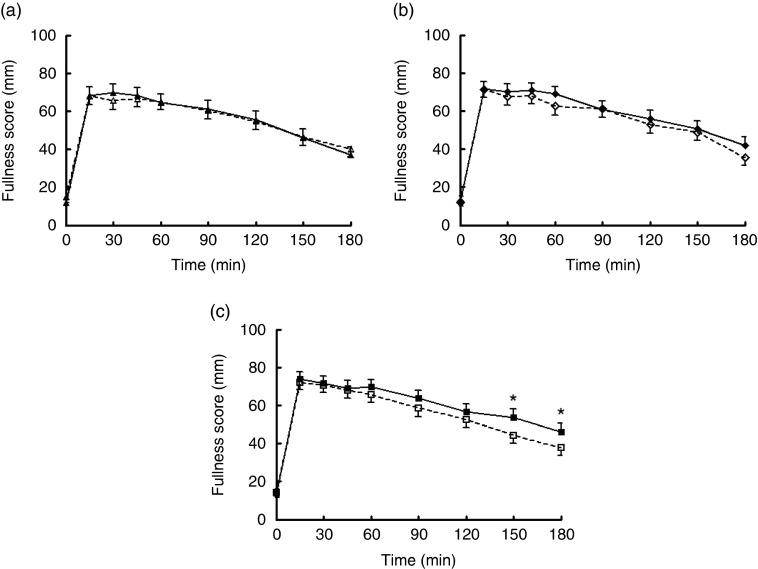
Fullness ratings over time at low (a), medium (b) and high (c) protein contents following the consumption of mycoprotein and chicken. Values are means, with standard errors. * *P*≤0·05 on repeated-measures linear mixed model and *post hoc* comparisons with Bonferroni’s correction. 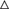, Low chicken; 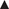, low mycoprotein; 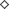, medium chicken; 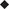, medium mycoprotein; 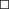, high chicken; 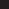, high mycoprotein.

As expected, there was a significant effect of time on all appetite ratings. Hunger, desire to eat and prospective food intake mean ratings significantly decreased following the consumption of the test meal between 0 and 15 min and thereafter increased slowly. Conversely, fullness significantly increased following the consumption of the test meal and slowly decreased throughout the following 3 h (Fig. 2). There were no differences in sickness ratings following the consumption of mycoprotein compared with chicken.

There were no significant differences in hunger, desire to eat and prospective food intake ratings at any time points between mycoprotein and chicken. There was a trend towards increased fullness following the consumption of mycoprotein compared with chicken (*P*=0·066). Post-test showed a significant effect of the treatment at the high-protein content at 150 min (chicken 46 (sd 4), mycoprotein 55 (sd 4), *P*=0·03) and 180 min (chicken 39 (sd 4), mycoprotein 47 (sd 4), *P*=0·04) (Fig. 2(c)).

Consistent with our results on energy intake from part A, there was no effect of the protein content *per se* on any of the appetite ratings overall or in mycoprotein and chicken separately.

#### Energy intake at *ad libitum* meal

**Fig. 3:**
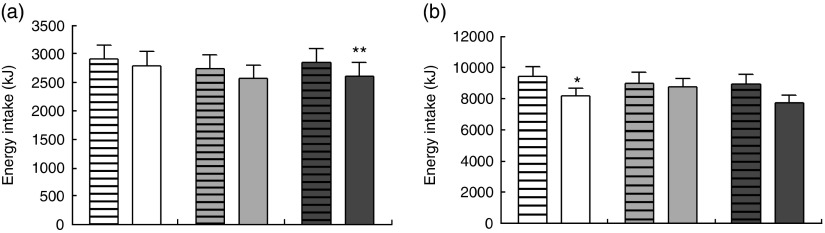
Energy intake at *ad libitum* meal (a) and during the following 24 h (b). Values are means, with standard errors represented by bar charts. * *P*≤0·05, ** *P*≤0·01 difference between mycoprotein and chicken analysed with repeated-measures linear mixed model and *post hoc* comparisons with Bonferroni’s correction. 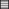, 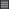, 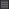, Chicken; 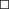, 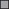, 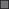, mycoprotein; 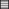, low protein; 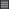, medium protein; 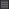, high protein.

The energy intake at the *ad libitum* lunch following the different test meals is shown in Fig. 3(a). There was a significant effect of the type of protein (mycoprotein *v.* chicken) on energy intake (*P*=0·008): overall, mycoprotein significantly decreased energy intake at the *ad libitum* meal further than the chicken test meal (chicken 2657 (sd 155) kJ (635 (sd 37) kcal), mycoprotein 2494 (sd 155) kJ (596 (sd 37) kcal)). Post-tests showed that energy intake following the high-mycoprotein meal was 10 % (280 kJ (67 kcal)) lower than following the chicken test meal (mean energy intake (EI) high chicken: 2711 (sd 234) kJ (648 (sd 56) kcal), mean EI high mycoprotein: 2431 (sd 209) kJ (581 (sd 50 kcal)), *P*=0·009). Differences in energy intake between chicken and mycoprotein at the low and medium levels were not significant.

The content of protein did not have any significant effect on energy intake: a higher content of protein did not induce a greater reduction in energy intake compared with the low and medium contents of protein overall, or for chicken and mycoprotein separately.

As expected, there was a significant effect of sex on energy intake (*P*<0·001), with males eating significantly more than females.

Participants found the mycoprotein significantly less pleasant than the chicken meal. However, mean palatability ratings remained above 60 out of 100 for both meals (mean palatability score chicken: 71 (95 % CI 64, 77), mycoprotein: 65 (95 % CI 59, 72), *P*=0·023).

#### 24-h energy intake

There was a significant effect of the type of protein on energy intake at 24 h (*P*=0·027): mean energy intake over 24 h was reduced by 787 kJ (188 kcal) (9 %) following the consumption of mycoprotein compared with chicken (chicken: 9209 (95 % CI 8351, 10071) kJ (2201 (95 % CI 1996, 2407) kcal), mycoprotein: 8422 (95 % CI 7552, 9288) kJ (2013 (95 % CI 1805, 2220) kcal)) (Fig. 3(b)). Post-tests showed a significant difference at the low content (*P*=0·047) and approaching significance at the high content (*P*=0·083).

Similarly to the acute energy intake results, there was no significant effect of the protein content *per se* on energy intake over 24 h overall, or in mycoprotein and chicken separately.

#### Peptide tyrosine-tyrosine and glucagon-like peptide-1 concentrations

**Fig. 4:**
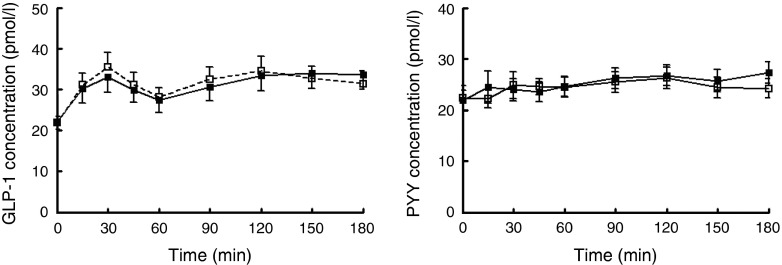
Plasma concentrations of glucagon-like peptide-1 (GLP-1) and peptide tyrosine-tyrosine (PYY) following the consumption of mycoprotein and chicken. Values are means, with standard errors. 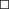, High chicken; 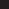, high mycoprotein.

Plasma GLP-1 and PYY concentrations following the consumption of the high-protein chicken and mycoprotein test meals are shown in Fig. 4.

There was a significant effect of time on GLP-1 concentrations (*P*<0·001). Following the consumption of the test meal, GLP-1 concentrations increased then decreased. PYY did not show such a pronounced postprandial excursion.

No significant effect of the protein type was observed on GLP-1 and PYY concentrations.

#### Glucose and insulin concentrations

**Fig. 5:**
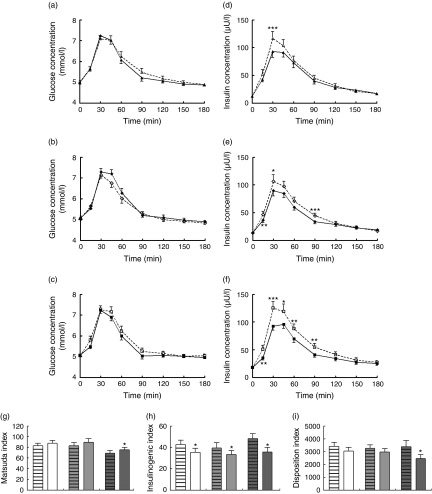
Plasma glucose and serum insulin concentrations at low, medium and high-protein contents and insulin sensitivity following the consumption of mycoprotein and chicken. (a–c) Plasma glucose concentrations at low (a), medium (b) and high (c) protein contents. (d–f) Serum insulin concentrations at low (d), medium (e) and high (f) protein contents. * *P*≤0·05, ** *P*≤0·01, *** *P*≤0·001 difference between mycoprotein and chicken analysed by repeated-measures linear mixed model and *post hoc* comparisons at each time point with Bonferroni’s correction. (g–i) Matsuda (g), Insulinogenic (h) and Disposition (i) indices. Values are means, with standard errors and log-transformed before analysis. * *P*≤0·05 difference between mycoprotein and chicken analysed by repeated-measures linear mixed model and *post hoc* comparisons with Bonferroni’s correction. 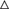, Low chicken; 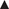, low mycoprotein; 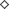, medium chicken; 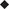, medium mycoprotein; 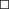, high chicken; 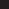, high mycoprotein; 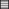, 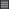, 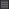, chicken; 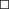, 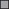, 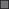, mycoprotein; 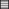, low protein; 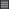, medium protein; 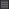, high protein.

Fig. 5(a–c) shows baseline and postprandial serum concentrations of glucose at low, medium and high protein contents over 180 min. As expected, there was a significant effect of time on glucose concentrations (*P*<0·001). Following the consumption of all test meals, glucose concentrations increased to reach a maximum at 30 min and thereafter returned to baseline concentrations. There was no significant effect of the type and level of protein on glucose concentrations at any time point. Fig. 5(d–f) shows baseline and postprandial serum concentration of insulin at low, medium and high protein content over 180 min. There was a significant effect of time on insulin concentrations (*P*<0·001). Insulin concentrations increased following the consumption of the test meal to reach a maximum at 30–45 min and returned to baseline within 2 h.

There was a significant effect of the time×type of protein interaction (*P*=0·002) on insulin serum concentrations: mycoprotein significantly reduced insulin concentration compared with chicken.

At the high-protein content, mycoprotein significantly reduced insulin concentrations compared with chicken by 41 % at 15 min, 27 % at 30 min, 20 % at 45 min, 21 % at 60 min and 26 % at 90 min. At the medium level of protein, mycoprotein reduced insulin concentrations compared with chicken by 22 % at 15 min, 12 % at 30 min, 12 % at 45 min, 13 % at 60 min and 24 % at 90 min. There was no significant effect of the protein content on insulin concentrations at other time points.

Analysis of the incremental AUC (IAUC) showed a significant effect of the type of protein overall (*P*=0·004) (Table 4). Post-tests showed a significant reduction in insulin IAUC of 8 % at low (*P*=0·024), 12 % at medium (*P*=0·001) and 21 % at high content of protein (*P*<0·001).

**Table 4:** Incremental AUC for glucose and insulin (Mean values with standard errors; geometric means with 95 % confidence interval adjusted for age)

	Low					Medium					High				
	C		MP			C		MP			C		MP		
	Mean	sem	Mean	sem	*P*	Mean	sem	Mean	sem	*P*	Mean	sem	Mean	sem	*P*
Glucose IAUC (mmol/min per litre) Insulin IAUC (μU/min per litre)	1019	13	1006	14	NS	983	13	992	9	NS	1013	17	976	14	NS
Mean	10 364^a,b^		9481		0·024	9650^a^		8510		0·001	11 178^b*^		8884		<0·001
95 % CI	8837, 11 891		7954, 11 008			8571, 10 728		7431, 9588			9604, 12 752		7311, 10 458		

C, chicken; MP, mycoprotein; IAUC, incremental AUC. ^a,b^ Mean values with unlike superscript letters were significantly different. * *P* values and letters correspond to mixed model post-test comparisons between low, medium and high protein content for each treatment and are given with Bonferroni’s correction (*P*=0·029).

There was a significant effect of the protein content on insulin IAUC (*P*=0·005). Post-tests showed that the effect of the protein content was significant for chicken (*P*=0·016) but not for mycoprotein (Table 4). The high level of chicken induced significantly higher insulin concentrations compared with the medium level, with no significant difference between low and medium and low and high levels.

#### Insulin sensitivity

Matsuda, Insulinogenic and Disposition Indices, shown in Fig. 5(g–i), were log-transformed before analysis. Overall, there was a significant effect of the type of protein on Matsuda Index (*P*=0·026), Insulinogenic Index (*P*=0·001) and Disposition Index (*P*=0·007). Mycoprotein improved insulin sensitivity compared with chicken, as shown by a significantly higher Matsuda Index (chicken: 78 (95 % CI 70, 86), mycoprotein: 84 (95 % CI 76, 92), *P*=0·026 for log-transformed data). Post-tests at each level showed a significant difference between mycoprotein and chicken at the high-protein content (high chicken: 64·0 (95 % CI 54·7, 74·8), high mycoprotein: 70·9 (95 % CI 62·0, 81·0), *P*=0·041 for log-transformed data) but not at the medium and low levels. Mycoprotein significantly reduced the Insulinogenic Index by 21 % compared with chicken (Insulinogenic Index chicken: 43 (95 % CI 37–48), Insulinogenic Index mycoprotein: 34 (95 % CI 29–39), *P*=0·001 for log-transformed data). Post-tests showed that mycoprotein reduced the Insulinogenic Index compared with chicken by 18 % at the low-protein content (low chicken: 38 (95 % CI 31, 47), low mycoprotein: 31 (95 % CI 25, 39), *P*=0·011), 15 % at the medium-protein content (medium chicken: 33 (95 % CI 25, 43), medium mycoprotein: 28 (95 % CI 22, 37), *P*=0·09) and 30 % at the high-protein content (high chicken: 43 (95 % CI 36, 53), high mycoprotein: 30 (95 % CI 23, 39), *P*=0·006). Mycoprotein significantly reduced the Disposition Index by 16 % compared with chicken (Disposition Index chicken: 3355 (95 % CI 2944, 3766); Disposition Index mycoprotein 2832 (95 % CI 2421, 3244), *P*=0·007 for log-transformed data). Post-tests showed significant differences between mycoprotein and chicken at the high-protein content (high chicken: 3392 (95 % CI 2398, 4384), high mycoprotein: 2461 (95 % CI 1903, 3020), *P*=0·028) but not at medium and low protein contents.

There was a significant effect of the content of protein on the Matsuda Index (*P*<0·001). Post-test within protein types showed a significant effect of the protein content in both mycoprotein (*P*=0·017) and chicken (*P*=0·002). The Matsuda Index following the high-protein content was significantly lower compared with that from the low-protein content for chicken (high chicken: 64 (95 % CI 55, 65), low chicken: 78 (95 % CI 68, 90), *P*=0·012) and almost significantly lower for mycoprotein (high mycoprotein: 71 (95 % CI 62, 81), low mycoprotein 83 (95 % CI 72, 95), *P*=0·059).

### Part B: gastric emptying, energy expenditure and substrate oxidation study

A total of fourteen volunteers (nine females and five males, mean age: 37·2 (sd 4·5) years, mean BMI: 28·4 (sd 0·6) kg/m^2^) completed the study. Baseline characteristics are shown in Table 3.

#### Appetite ratings

**Fig. 6:**
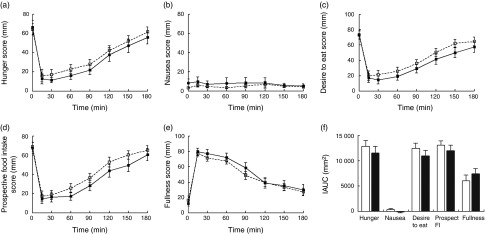
Appetite ratings following the consumption of mycoprotein and chicken: hunger (a), nausea (b), desire to eat (c), prospective food intake (d), fullness (e) and IAUC for all appetite ratings (f). Values are means, with standard errors. IAUC, incremental AUC. 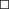, Chicken; 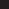, mycoprotein.

Similarly to results from part A, there was a significant effect of time (*P*<0·001) on all appetite ratings apart from sickness (Fig. 6).

There was no significant effect of mycoprotein on hunger, sickness, desire to eat, prospective food intake and fullness at any time point compared with chicken. This was confirmed by the overall analysis of the IAUCAUC showing no significant effect of the protein type (Fig. 6).

#### Glucose and insulin concentrations

In line with previous findings from part A, there was no significant difference in plasma glucose concentrations following the consumption of mycoprotein and chicken (IAUC glucose chicken: 1006 (sd 95), IAUC glucose mycoprotein: 1004 (sd 92), NS).

Analysis of the insulin IAUC showed that insulin concentrations were significantly lower following the consumption of mycoprotein than following the consumption of chicken (IAUC insulin chicken 10693 (sd 4117), IAUC insulin mycoprotein: 9288 (sd 3076), *P*=0·015).

#### Gastric emptying

**Fig. 7:**
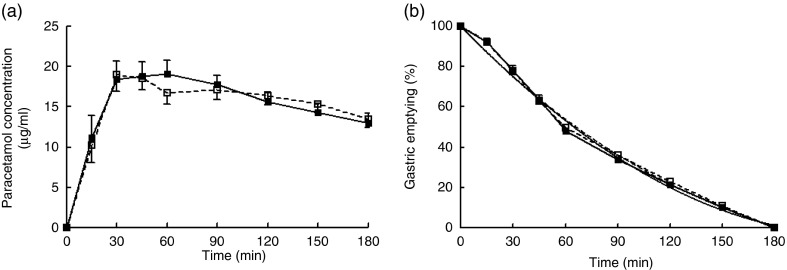
Serum paracetamol concentrations (a) and gastric emptying rate (b) following the consumption of mycoprotein and chicken. (a) Values are means, with standard errors. (b) Values are mean gastric emptying percentages for all participants with a non-linear fit of the curve. 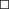, Chicken; 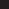, mycoprotein; 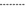, non-linear fit (chicken); 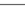, non-linear fit (mycoprotein).

There was no difference in paracetamol concentrations (Fig. 7(a)) or percentage gastric emptying (Fig. 7(b)) over time between mycoprotein and chicken. The T50 was 65 min for mycoprotein and 66 min for chicken (NS).

As expected, there was a significant effect of time (*P*<0·001) on paracetamol concentrations, with paracetamol concentrations increasing rapidly following the consumption of the test meal to reach a maximum and slowly decreasing over the following 3 h (Fig. 7(a)).

There was no difference in paracetamol concentrations at any time point following the consumption of mycoprotein or chicken (Fig. 7(a)). Analysis of the AUC showed no significant differences in paracetamol concentrations between mycoprotein and chicken overall. No significant differences in time-to-peak were observed.

#### Resting energy expenditure and substrate oxidation

**Fig. 8:**
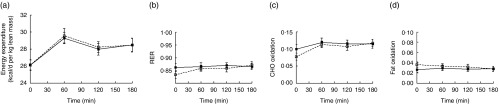
Energy expenditure (per kg lean mass) (a), RER (b), carbohydrate oxidation (c), fat oxidation (d) following the consumption of mycoprotein and chicken. Values are means, with standard errors. CHO, carbohydrate. 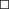, Chicken; 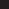, mycoprotein.

There was a significant effect of time on REE (*P*<0·001). REE increased following the consumption of the test meal. No significant difference in REE was observed following the consumption of mycoprotein compared with chicken (Fig. 8(a)).

RER, carbohydrate oxidation (OX^CHO^) and fat oxidation (OX^FAT^) are shown in Fig. 8(b–d). There was no significant effect of time on the RER (Fig. 8(b)) and the OX^FAT^ (Fig. 8(d)). Analysis of the OX^CHO^ showed a significant effect of time (*P*<0·001), with OX^CHO^ increasing following consumption of the test meal (Fig. 8(c)).

The analysis showed no significant effect of the protein type on RER, OX^CHO^ and OX^FAT^ at any time points (Fig. 8).

### Metabonomics analysis

**Fig. 9:**
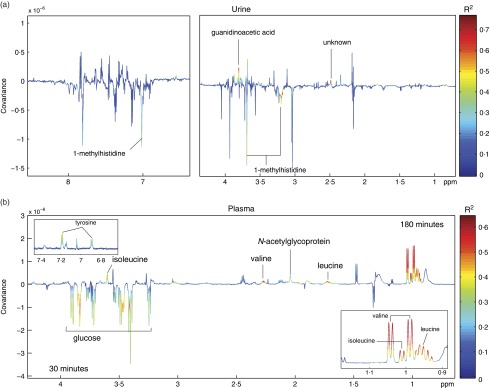
Orthogonal projection to latent structures-discriminant analysis models comparing the urinary metabolic profiles of volunteers following chicken and mycoprotein intake (a) and the plasma metabolic profiles 30 *v.* 180 min after mycoprotein consumption (b). Colour corresponds to the correlation of the metabolites to class discrimination ((a) mycoprotein *v*. chicken; (b) 30 *v*. 180 min post-mycoprotein consumption). Colour indicates the strength of correlation. *N*-acetylcarnitine, carnitine and anserine increase following chicken intake. Guanidinoacetic acid and unknown (2·48) increase following mycoprotein intake. Valine, isoleucine, leucine, and *N*-acetyl-glycoprotein increase at 180 min. Glucose decreases at 180 min. ppm, Parts per million.

An OPLS-DA model with good predictive ability (Q^2^Y=0·60) was obtained comparing the urinary metabolic profiles of volunteers following chicken and mycoprotein intake (Fig. 9(a)). Mycoprotein consumption resulted in the greater excretion of guanidinoacetic acid (GAA), whereas excretion of 1-methylhistidine was observed with chicken intake. An unknown metabolite (*δ* 2·48 (singlet)) was also excreted in greater amounts following mycoprotein ingestion compared with the chicken diet.

An OPLS-DA model was constructed to compare the plasma metabolic profiles 30 *v.* 180 min after mycoprotein consumption (Fig. 9(b); Q^2^Y=0·6). This model was consistent with the plasma glucose measures showing blood glucose to be higher at 30 min than at 180 min. At the later time point, the branched-chain amino acids, leucine, isoleucine and valine, were found to be higher in the blood. *N-*acetylglycoproteins, markers of inflammation, were also present at higher amounts 180 min post mycoprotein consumption compared with the earlier sampling point.

**Fig. 10:**
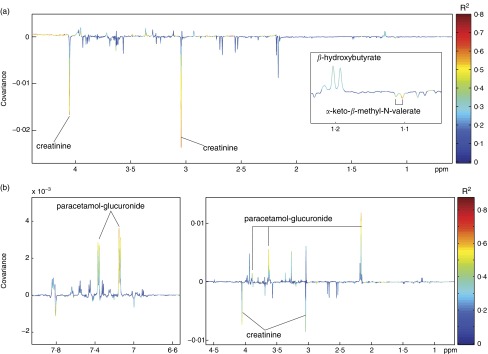
Orthogonal projection to latent structure models showing metabolic variation associated with fullness following mycoprotein (a) and chicken (b) intake. (a) Colour indicates the correlation of the metabolites with fullness. After mycoprotein intake, creatinine and the deamination product of isoleucine, *α*-keto-*β*-methyl-*N*-valerate, were inversely related to fullness and *β*-hydroxybutyrate was positively associated. (b) Significant associations are shown in red. After chicken intake, paracetamol-glucuronide was positively associated with fullness and creatinine was negatively associated.

Separate OPLS models were constructed to identify metabolic variation associated with fullness following mycoprotein (Fig. 10(a)) and chicken (Fig. 10(b)) intake. Significant models were returned for both diets (chicken Q^2^Y=0·475; mycoprotein Q^2^Y=0·429), but variation was observed in the metabolites associated with fullness. Following chicken intake, paracetamol-glucuronide was positively associated with fullness, whereas creatinine was negatively associated. After mycoprotein consumption, creatinine and the deamination product of isoleucine, *α*-keto-*β*-methyl-*N*-valerate, were inversely related to fullness, whereas the ketone body, *β*-hydroxybutyrate, was positively associated.

## Discussion

We hypothesised that mycoprotein would reduce the energy intake in overweight and obese individuals in a dose-dependent manner, and this would be mediated by changes in appetite regulating GI hormone concentrations. Confirming previous findings in lean individuals^(^ ^21^ ^,^ ^22^ ^)^, this is the first study showing that the consumption of 132 g of mycoprotein by healthy overweight and obese adults reduces energy intake by 10 % at an *ad libitum* meal compared with a macronutrient-matched meal containing chicken. Low and medium quantities of mycoprotein did not reduce energy intake, suggesting that a minimum amount of mycoprotein is needed to affect appetite. If mycoprotein was consumed regularly and its effect maintained over the long term, this 10 % reduction in energy intake may represent a significant weight loss. The present study also showed that mycoprotein reduced energy intake by a further 9 % over 24 h compared with chicken; however, a significant effect was only observed at the lower content of protein. Although we do not have a clear explanation for this observation, it is possible that the study was underpowered to look at differences in 24-h energy intake or that underestimation or poor completion of the food diaries, which are both common in overweight human volunteers, increased the error in measurement of energy intake. In line with previous findings by Burley *et al.* ^(^ ^21^ ^)^, no significant effects of mycoprotein consumption on appetite ratings were noted. Although the use of VAS is common in appetite studies, the sample size needed to detect significant small differences in appetite ratings after statistical correction for multiple comparisons was possibly not reached in this study.

We found that the protein content of the meal (low, medium, or high) did not have any significant effect *per se* on energy intake at the *ad libitum* meal. Although previous studies have suggested a negative dose–response in appetite to increasing contents of protein, differences in protein content in those studies (13–26 g in the study by Astbury *et al*.^(^ ^34^ ^)^) were greater than in our study (6–10 g)^(^ ^33^ ^–^ ^35^ ^)^. Consequently, the differences in protein content in our study may not have been sufficient to induce significant reductions in energy intake. Previous studies using greater differences in protein intake also showed no effect on appetite ratings^(^ ^34^ ^)^.

The investigations of the mechanisms underlying the mode of action of mycoprotein were inconclusive. Part A of our study showed that mycoprotein did not induce a significant increase in GLP-1 and PYY plasma concentrations compared with the chicken meal, suggesting that the reduction in energy intake is not mediated by changes in the release of these hormones. Furthermore, although there was an increase in GLP-1 concentrations following the consumption of the test meal in our study, no postprandial increase in PYY concentrations could be detected, suggesting that the energy load of the meal was not sufficient to induce a detectable increase in PYY. Other GI hormones may have played a role in the energy intake reduction following mycoprotein intake. Further studies should, for example, investigate the role of cholecystokinin, which has been shown to be associated with gastric distension and to play a role in appetite regulation.

Using the paracetamol method, no significant differences in gastric emptying following the consumption of the mycoprotein and chicken test meals were found. The paracetamol method can be used as a proxy to estimate gastric emptying rate in solid meals, as it is a relatively inexpensive replacement to the scintigraphic, polyethylene glycol dilution and ^13^C acetate breath test methods^(^ ^36^ ^,^ ^37^ ^)^. Consistent with previous studies, paracetamol was supplied to the participants in a liquid form. However, participants consumed the paracetamol drink along with their meal in our study, whereas paracetamol was given at the end of a solid meal in previous studies. Although participants were asked to keep a similar constant drinking pace during both visits, it is possible that because mycoprotein is bulkier the rate of drinking was faster than during the chicken test meal. This may be clarified by mixing paracetamol directly within the test meal in future studies. Alternatively, mycoprotein may modulate gastric distension or intestinal transit time without affecting gastric emptying, as its energy density is lower than that of chicken.

In the second part of our study, mycoprotein did not induce significant changes in energy expenditure. Matching of the test meals for protein content may explain this observation, as protein has been shown to increase energy expenditure independently of the energy content in previous studies^(^ ^38^ ^)^. Changes in RER and substrate oxidation were not significant. As this area of research is relatively new, there are no studies directly investigating the effect of fibre supplementation on substrate oxidation. The amount of fibre contained in mycoprotein may not be sufficient to induce acute changes in substrate oxidation. Whether prolonged supplementation of mycoprotein could induce significant alterations in substrate oxidation over the long term is unknown, but it is unlikely that this would be the main mechanism of action of mycoprotein.

The main difference between the test meals resulted from the fibre content. The fibre contained in mycoprotein, composed of chitin and *β*-glucan linked in a strong matrix, is 88 % insoluble. This suggests that the insoluble fibre may be the main active compound in mycoprotein. Chitin is generally absent from the diet of humans, and its impact in the GI tract is poorly understood. It may be resistant to digestion, as its structure is similar to that of cellulose and xylose. The largely insoluble *β*-glucan in mycoprotein is different from soluble plant-derived *β*-glucan originating from barley and oats, which have been shown to reduce energy intake, act on intestinal transit and glucose homoeostasis and modulate GI hormone release in several animal and human studies^(^ ^12^ ^–^ ^14^ ^,^ ^39^ ^)^. It is possible that, although of different structure and properties, mycoprotein *β*-glucan presents common characteristics and acts in a similar way. Although there have been few studies that have investigated the digestion of mycoprotein in the GI tract^(^ ^40^ ^,^ ^41^ ^)^, its effects on appetite regulation remain to be explored. It would be interesting to extract digested mycoprotein from the GI tract of animals and analyse its composition at regular time intervals following oral ingestion.

Our study found that mycoprotein did not induce any significant changes in glycaemic response compared with chicken, which is consistent with findings by Marks *et al*.^(^ ^42^ ^,^ ^43^ ^)^. Although Turnbull *et al*. found a significant reduction in glucose concentration at 60 min following the consumption of mycoprotein compared with a soya meal, the design of their study differed considerably, because they used a liquid oral glucose tolerance test-like meal containing soya, milk and mycoprotein powder. Consistent with previous findings, our study showed that mycoprotein reduced postprandial serum insulin concentrations by 8–21 % compared with chicken in overweight and obese individuals^(^ ^20^ ^)^. Furthermore, analysis of the Matsuda, Insulinogenic and Disposition Indices suggested that mycoprotein improves insulin sensitivity and reduces *β*-cell output without altering glucose concentrations. More robust measurements of insulin sensitivity assessment, such as the hyperinsulinaemic euglycaemic clamp, should be used to confirm whether mycoprotein results in the sparing of the *β*-cells following a long-term intervention. Our hypothesis is that the structure of mycoprotein itself plays a role in the regulation of glucose metabolism following oral ingestion by limiting carbohydrate absorption in the intestine. Native mycoprotein paste is composed of *β*-glucan-chitin filaments of various lengths randomly oriented in all directions and bound by a gel matrix^(^ ^44^ ^)^. In a previous study investigating the impact of the length of mycoprotein filaments, Marks found that 90g of mycoprotein containing long filaments delayed gastric emptying compared with the same quantity of mycoprotein containing short filaments^(^ ^43^ ^)^. Although no difference in gastric emptying was found in our study, it does imply that the structure and length of the filaments play a role in the digestion of mycoprotein.

To further understand the mechanism through which mycoprotein modulates appetite regulation, the urinary and plasma metabolic profiles were studied across the participants. Multivariate statistical analysis of the urinary profiles identified that 1-methylhistidine was excreted in greater amounts following consumption of chicken. This metabolite derives from the breakdown of anserine and has been previously reported as a biomarker of meat consumption. Indeed, Sjolin *et al.* ^(^ ^45^ ^)^ have shown urinary 1-methylhistidine to be associated with chicken ingestion.

Following mycoprotein consumption, a greater excretion of GAA was observed compared with chicken intake. GAA is a methyl-group acceptor and the precursor for creatine; as such, it is an essential substrate for muscle energy metabolism. It can be synthesised endogenously from arginine and glycine in the liver and kidneys. Although there is lack of data on the role of GAA in humans, dietary GAA has the potential to spare dietary arginine. Recently, there is preliminary evidence that arginine may play a role in appetite regulation^(^ ^46^ ^,^ ^47^ ^)^. Another metabolite was excreted in greater amount following mycoprotein ingestion, although we were unable to identify it in the context of this study. In response to the mycoprotein diet, it is a possible to speculate that the ketone body *β*-hydroxybutyrate was found to be positively associated with fullness. This was not observed in response to the chicken diet. *β*-Hydroxybutyrate has been previously linked to appetite suppression through central effects at the hypothalamus. *β*-Hydroxybutyrate is normally associated with long-term fasting; in the case of this study, the volunteers were in a fed state. At present, the unique association between *β*-hydroxybutyrate and fullness with the mycoprotein diet remains unclear. It does raise an interesting possible explanation for the appetite suppression, which would be worthy of further study.

### Conclusion

Our results demonstrate for the first time in overweight and obese volunteers that mycoprotein acutely reduces energy intake and improves glycaemic profile. Our study was not able to provide a clear mechanistic explanation, although the metabonomics analysis did identify candidate biomolecules that warrant further investigation. There is need of longer-term studies to investigate the potential of mycoprotein in the prevention of obesity and T2DM.
